# Change from lung adenocarcinoma to small cell lung cancer as a mechanism of resistance to afatinib

**DOI:** 10.18632/oncotarget.17607

**Published:** 2017-05-04

**Authors:** Paolo Manca, Marco Russano, Francesco Pantano, Giuseppe Tonini, Daniele Santini

**Affiliations:** ^1^ Department of Medical Oncology, University Campus Bio-Medico, Rome, Italy

**Keywords:** afatinib resistance, EGFR-TKI resistance, EGFR inhibitor resistance, small cell lung cancer switch, tumor switch

## Abstract

We report the case of a patient affected by advanced EGFR mutation-positive lung who experienced resistance to therapy during treatment with Afatinib through the occurrence of a switch of tumor histotype to small cell lung cancer (SCLC) with features of a G3 neuroendocrine carcinoma. Unexpectedly, the switch to SCLC histotype occurred in the only site not responsive to afatinib and subsequently the most responsive to chemotherapy. Our case shows that occurrence of switch to SCLC is a possible mechanism of resistance during treatment with Afatinib.

## BACKGROUND

Survival of patients with NSCLC changed sharply after introduction of epidermal-derived growth factor receptor tyrosine kinase domain inhibitors of (EGFR-TKIs). However, despite the great effectiveness of these therapies, patients often experience relapse of the disease. Great efforts are being made to identify mechanisms of resistance in order to improve management of these patients. The identification of T790M mutation and the subsequent development of mutation-specifically targeted EGFR TKIs is the most valid example.

Among the different mechanisms of resistance to EGFR-TKIs is the switch to SCLC histotype. Different cases of such switch have been reported and have already been summarized by Shi-Yu Jiang et al [1]. Nevertheless the switch to SCLC is a known mechanism of resistance to treatment with gefitinib and erlotinib and no cases of switch were reported to occur during treatment with Afatinib. We report a case of a resistance occurred through a switch to neuroendocrine histotype in a patients with adenocarcinoma of the lung during treatment with Afatinib.

## CASE PRESENTATION

The patient is a 74 years old white woman who came to our attention in December 2013 lamenting fatigue and dry cough in the past month. She was not a present nor a former smoker and didn't refer any relevant comorbidity. The patient brought to our attention:

- a thorax-abdomen-pelvis CT scan without iodine based contrast agent from November 2011 showing in the right inferior lobe a 57×35x48 mm new solid mass with irregular borders
- a lumbo-sacral MRI scan showing multiple bone lesions of the dorsal vertebrae

In January 2014, the patient underwent a CT scan guided biopsy of lung lesion showing cytomorphologic aspect of NSCLC; the immunohistochemistry analysis showed positivity for TTF1, local and weak positivity for synaptophysin and chromogranin and negativity for p63 expression thus deposing for adenocarcinoma histotype. Molecular analysis with Pyrosequencing showed deletion of exon 19 in the EGFR gene; analysis with fluorescent in-situ hybridization (FISH) showed no ALK gene rearrangement.

The patient has therefore carried out a total body CT scan with iodine contrast agent for systemic stadiation showing in the inferior right lobe a 57×35x48 mm solid mass with irregular borders; multiple satellite nodules in the homolateral lung; multiple mediastinal nodes enlargement; new nodular formation of 15 mm in the right adrenal gland; multiple sclerotic lesions of dorsal and lumbar vertebrae, right clavicle, sternum, third right rib, fourth right and left rib, ilium and sacrum. Clinical staging was T4N3M1.

**Figure 1:**
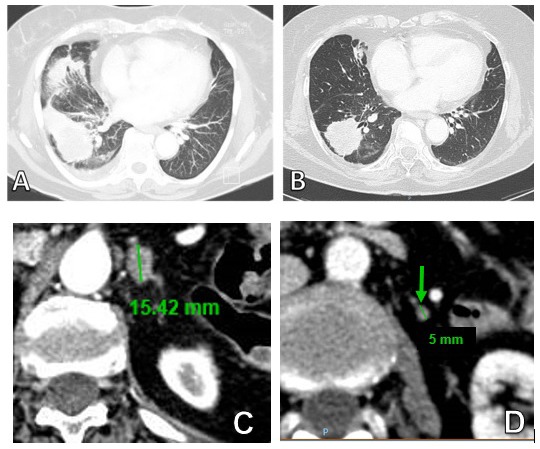
Thoracic CT scan of primary lesion (**A**) at baseline and (**B**) after 60 day of treatment with Afatinib. (**C**) Adrenal metastasis at baseline (**D**) and response to treatment after 60 days.

Afterwards in 03/18/2014 the patient started treatment with Afatinib tablets 40 mg/die every day. She had a good performance status at the beginning of treatment (ECOG PS 0). The patient suspended Afatinib after 40 days of treatment because of a G3 hand/foot syndrome that was successfully managed with topical steroid treatment and antibiotics. The patient resumed Afatinib tablets treatment nine days later with dose reduction to 30 mg/die every day. The patient continued treatment for further 20 days after whom she underwent total body CT scan with iodine contrast agent for systemic evaluation (05/06/2014). CT scan showed size reduction of right inferior lobe mass (55×41 *vs* 57×55 mm); size reduction of nodular formations in the homolateral lung; size reduction of involved nodes; size reduction of adrenal gland mass (5 *vs* 15 mm); stability of bone disease (Figure 1).

Since the treatment showed to be effective, the patient continued Afatinib 30 mg/day. After further 69 days of treatment the patient underwent a total body CT scan with iodine contrast agent (08/04/2014) that showed stability of all the site of disease.

The patient continued Afatinib treatment for further 70 days after whom underwent total body CT scan with iodine contrast agent for systemic evaluation (10/14/2014) that showed: stability of disease in mediastinal nodes, adrenal gland and bones; moderate enlargement of right inferior lobe mass (55×46 *vs* 55 × 41 mm); 3 new hepatic lesions in the II segment (4mm), the V segment (10 mm) and the VI segment (5mm) with metastatic appearance.

The clinical condition of the patient was stable, even treatment with afatinib had led clinical benefit resulting to the resolution of the cough. Therefore we decided to continue treatment with afatinib waiting to determine the extent of liver progression.

The patient resumed Afatinib tablets treatment, even though with a further dose reduction - 20 mg/die every day - because of previous hand/foot toxicity.

The patient underwent hepatic MRI scan with contrast agent (11/11/2014) that showed evident hepatic progression: 5 hepatic masses were described, one in II segment (9 mm *vs* 4 mm at the CT scan), one in the V-VIII segment (15 mm *vs* 10 mm at the CT scan), on in the VI segment (7 mm *vs* 5 mm at the CT scan), one in the VII segment (10 mm) and one in IV-VIII segment (14 mm).

We thus planned a hepatic biopsy on new lesions to assess whether a T790M mutation in the EGFR gene was present or not. The US guided hepatic biopsy took place in an inpatient regimen (11/21/2014); no complications occurred during the procedure. Unexpectedly, the biopsy revealed the presence of a small cell lung carcinoma with neuroendocrine differentiation (G3) with positivity for TTF1 and synaptophysin, focal positivity for CD56 and negativity for chromogranin. Molecular analysis showed persistence of the deletion of exon 19 of the EGFR gene. On the contrary, the T790M mutation was not found.

**Figure 2:**
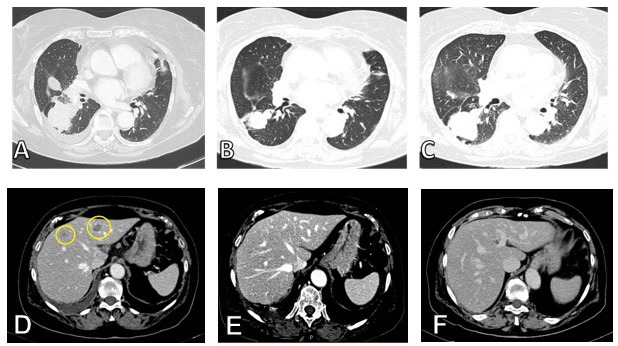
**A.** Thoracic CT scan of primary lesion before treatment with CBCDA + VP -16, **B.** Response after 3 cycles of CBCDA + VP -16 and **C.** progression after 5 cycles of CBCDA + VP -16. **D.** Liver metastases before treatment with CBCDA + VP -16, **E.** complete response after 3 cycles and **F.** persistence of complete response after 5 cycles of CBCDA + VP -16.

When the liver biopsy showed a neuroendocrine differentiation, it was evident that treatment with afatinib would have been ineffective. We considered the possibility that there were two different cell clones: one with neuroendocrine differentiation and resistant to afatinib, the other potentially still sensitive to Afatinib. Since the small cell cancer is more aggressive compared to adenocarcinoma, we decided to stop treatment with afatinib and begin chemotherapy. Thus we started (12/17/2014) chemotherapy based on Carboplatin AUC 5 day 1 **plus** etoposide 100 mg/m^2^ days 1-3 every 21d. After 3 cycles (03/12/2015) the patient underwent a total body CT scan for systemic evaluation: the exam showed partial response of right inferior lobe mass, complete response of mediastinal nodes and hepatic lesions and stability of disease of adrenal gland and bone lesion. The patient underwent two further cycles of CBCDA + VP-16 regimen until 05/27/15, when she underwent CT scan with iodine contrast agent examination for clinical suspect of disease progression (worsening cough and asthenia). The CT showed progressive disease of right inferior lobe mass, stability of disease of bone and adrenal gland lesions, persistence of complete response of hepatic lesions (Figure 2).

**Figure 3:**
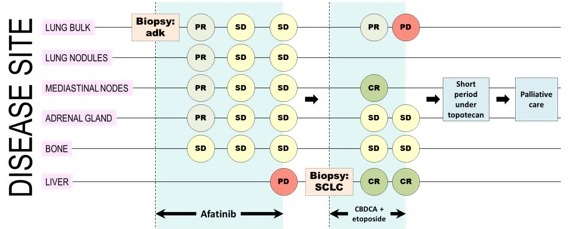
A representative image resuming disease course of the patient

The patient discontinued CBCDA + VP-16 regimen due to inefficacy and started in 06/12/2015 topotecan 4mg/m^2^ 1-8q21 schedule. She underwent CT scan with iodine contrast agent examination (08/20/2015) after 3 cycles of topotecan. The CT scan showed heavy progression of lung disease. Similarly. patient's clinical condition suddenly worsened (asthenia, dyspnoea, bone pain, dehydration, nausea). ECOG PS was 3. Then she was deferred to palliative care.

## DISCUSSION

This report showed a possible way of management and treatment in a peculiar setting of patients developing neuroendocrine differentiation at resistance to EGFR TKIs.

There are several documented mechanisms of resistance to EGFR TKIs. The most common mechanism of resistance in a 155 case series treated with erlotinib or gefitinib was occurrence of T790M mutation (60%) [2]. In the largest survey aimed to identify the mechanisms of resistance to afatinib, the T790M mutation was identified in about half of cases, however the small cell trasformation was not ever found [3].

This report thus fills a gap in previous knowledge concerning documented mechanisms of resistance to Afatinib. To our knowledge, this is the first case of resistance occurring during treatment with Afatinib whose mechanism is a switch from lung adenocarcinoma to SCLC histotype.

Popat et al. reported a similar case in which the patient was documented with an adenocarcinoma to SCLC switch after experiencing relapse after erlotinib therapy; similarly to our patient, Popat patient showed subsequent generalized sensitivity to CBDCA plus etoposide therapy. Therapy with Afatinib was tried in their patient before CBDCA plus etoposide regimen and immediately showed no benefit; however it should be noted that their patient was already resistant to an another EGFR-TKI. Also in their case no resistance mutations were found in the EGFR gene, suggesting that SCLC transformation is sufficient to develop resistance also to Afatinib therapy [4].

The clinical issue highlighted from this case is whether Afatinib therapy discontinuation is the best clinical choice in patient with responsive disease in all sites except the site of relapse. Our patient showed only hepatic progression while stability of the disease was maintained all over the other sites of disease localization. Most intriguingly, the hepatic lesion was the only site of disease showing complete response at the time of occurrence of CBDCA plus etoposide resistance.

Since data in the literature in this setting are lacking, we cannot know whether the continuation of therapy with Afatinib is a valid therapeutic option compared to chemotherapy. A randomized, open-label, phase III trial [5] demonstrated that continued treatment with afatinib with the additions of paclitaxel significantly improved ORR and PFS *vs* CT alone in heavily pretreated patients with acquired resistance to anti-EGFR therapies. However, no analysis taking into consideration the mechanism of resistance to EGFR TKIs were performed in this study.

In conclusion our case shows that, although the main mechanisms of resistance to EGFR TKIs have been clarified and new strategies have been developed to overcome them, there is still a need to clarify what is the best approach for each mechanism, especially for those patients who develop less common ones.
